# Full-length inhibitor protein is the most effective to perturb human dUTPase activity

**DOI:** 10.1038/s41598-025-86131-7

**Published:** 2025-02-09

**Authors:** Bianka Kőhegyi, Zoé S. Tóth, Enikő Gál, Máté Laczkovich, András Benedek, Beáta G. Vértessy, Kinga Nyíri

**Affiliations:** 1 Department of Applied Biotechnology and Food Science, Faculty of Chemical Technology and Biotechnology, Budapest University of Technology and Economics, Műegyetem rkp. 3, Budapest, 111 Hungary; 2 Institute of Molecular Life Sciences, HUN-REN Research Centre for Natural Sciences, Magyar tudósok krt 2, Budapest, 1117 Hungary; 3 Doctoral School of Biology, Institute of Biology, ELTE Eötvös Loránd University, Pázmány Péter sétány 1/A, Budapest, 1117 Hungary

**Keywords:** dUTPase, Proteinaceous inhibitor, Peptide inhibitor, Structure based design, X-ray crystallography, Structure determination, Enzymes, Hydrolases

## Abstract

It has been demonstrated recently that knockout of the dUTPase enzyme leads to early embryonic lethality in mice. However, to explore the physiological processes arising upon the lack of dUTPase an effective and selective enzyme inhibitor is much needed. A highly specific and strong binding proteinaceous human dUTPase inhibitor described by us recently was a promising starting point to develop a molecular tool to study temporal and conditional dUTPase inhibition *in cellulo*. Towards this end we determined the 3D crystal structure of the crystallizable amino terminal domain of inhibitor protein, named Stl^NT^ in complex with the human dUTPase and designed several point mutants based on the structure to improve the inhibition effectivity. The effect of Stl^NT^ and a peptide derived from the full-length inhibitor on the activity of the human dUTPase was also tested. We showed that the C-terminal part of the Stl protein omitted from the crystal structure has an important role in the enzyme inhibition as the full-length Stl is needed to exert maximal inhibition on the human dUTPase.

**Supplementary Information:**

The online version contains supplementary material available at 10.1038/s41598-025-86131-7.

## Introduction

The dUTPase enzyme plays a key role in preventive DNA repair by eliminating dUTP from the DNA biosynthetic pathway, meanwhile it also produces dUMP, a precursor for dTTP biosynthesis^1–3^. The lack of dUTPase activity leads to high cellular dUTP level, and increases the frequency of uracil incorporation into the genome upon synthesis or repair as most polymerases do not discriminate dUTP over dTTP^4,5^. Several DNA repair enzymes, namely uracil-DNA glycosylases target and cleave uracil from the DNA^6^. Although this mechanism is evolved to eliminate mutagenic U: G pairs resulting from cytosine deamination, in most cases it also acts on thymine replacing uracils. Overwhelming of the uracil base excision repair mechanism by perturbing the function of dUTPase induces DNA fragmentation and cell death. Based on this prominent role of the dUTPase enzyme it is in the focus of onco-therapeutic drug design projects and targeted to fight pathogenic microorganisms^7,8^.

Besides therapeutic applications, selective inhibitors are much needed to study the physiology of dUTPase enzyme in mammalian cell lines and organisms. It has been shown in case of mice that dUTPase knockout leads to an early embryonic lethal phenotype^9^, while knockout studies on dUTPases have not yet been succeeded for any human cell line. To better understand the processes emerging upon deficient dUTPase activity an effective and selective inhibitor would be necessary. Although there exist a potent small molecular human dUTPase inhibitor (TAS-114) that also inhibits dihydropyrimidine dehydrogenase, thus it is not suitable for mechanistic studies on dUTPase^10,11^.

Besides small molecular inhibitors, proteinaceous inhibitors provide a promising alternative for modulating enzyme function. Their potentially enhanced selectivity could decrease intracellular off-target effects. Such protein inhibitors may also present powerful tools for *in cellulo* studies on the effect of enzyme inhibition. It has been shown for example that a bacteriophage-related uracil-DNA glycosylase inhibitor can significantly reduce the enzymatic activity of the human enzyme *in vitro* and *in vivo*^12–15^.

In 2010 it was discovered that φ11 and 80α bacteriophage dUTPases interact with the staphylococcal Stl protein involved in the regulation of a *Staphylococcus aureus* pathogenicity island^16^. To understand the molecular mechanism of Stl-dUTPase interaction, we studied several dUTPase-Stl complexes with various biochemical and biophysical methods^17–22^. Based on our hydrogen deuterium exchange mass spectrometry (HDX-MS) results a short segment of Stl binds to the active site of φ11 phage and human dUTPase blocking the pocket from the substrate^17,18^. The same binding mode was confirmed by the crystal structure of a C-terminally truncated Stl construct with φ11 phage and shrimp dUTPases^23,24^. We revealed that Stl is also a potent inhibitor of the φ11 phage dUTPase^22,25^, and it has been shown that Stl protein also inhibits the *Mycobacterium tuberculosis*, *Drosophila melanogaster*, *L. vannamei* and human dUTPases although to different extent^17,19,20,24^. While the φ11 phage dUTPase is almost completely inactivated by Stl^22^, the human dUTPase retained 30% of its activity upon addition of Stl in large excess^17^.

In this work we set out to explore key aspects of human dUTPase (hDUT) inhibition by Stl and we aimed to engineer the Stl protein to enhance its inhibitory potency for hDUT. We followed two strategies to enhance the inhibitory effect of Stl on human dUTPase. On one hand, we determined the complex of hDUT with the crystallizable amino terminal domain of Stl (Stl^NT^) by X-ray crystallography. Based on analysis of this structure we designed point mutant full-length Stl proteins, with potentially enhanced affinity to human dUTPase. On the other hand, we introduced point mutations in the Stl protein, which impair its dimerization, thus it could be more effective in dUTPase inhibition. We also tested if an Stl-derived peptide or Stl^NT^ can act as an effective human dUTPase inhibitor.

## Methods

### Protein preparation

Plasmids encoding Stl mutants were created by using the NEB Q5 mutagenesis kit. Primers were designed by NEBaseChanger webtool (https://nebasechanger.neb.com/), from an original construct described in^22^. To gain constructs, which can be enzymatically biotinylated for the biolayer interferometry measurements a so-called Avi-tag (MSGLNDIFEAQKIEWHE) was introduced to the carboxy terminus of Stl^NT^ (residues 1-159) and Stl^CT^ (residues 175–267) proteins again using the NEB Q5 mutagenesis kit and NEBaseChanger webtool. While in case of dUTPase the gene encoding the human dUTPase was cloned into a PAN4 vector in frame with the N-terminal Avi-tag^26^. Validity of constructs were verified by sequencing.

### Protein expression and purification

Stl variants and human dUTPase proteins were expressed and purified as described in our previous work^17^. Biotinylated Stl constructs for BLI were expressed and purified as described in^26^, during the purification 50 µM biotin and a pellet of E. *coli* AVB101 cultured overnight at 18 °C in 0.5 L broth were added to the pellet of the target protein to include the biotinylating enzyme (BirA) in the lysate. Biotinylated human dUTPase was expressed in *E. coli* AVB101 cells in 500 ml LB supplemented with 100 µg/ml ampicillin and 10 µg/ml chloramphenicol at 18 °C overnight, 1.5 mM IPTG, 50 µM biotin was added at OD_600_ = 0.6. The peptide 95-DKMYSYVNKAYYNDGDIYYSSYD-117 used for inhibitory studies was ordered from GeneScript.

### Steady state dUTPase activity assay

The steady state dUTPase enzyme activity in the presence and absence of Stl variant was measured at 25 °C by following the proton release during the dUTP hydrolysis causing the change in the absorbance of phenol red pH indicator at 559 nm in the assay buffer (1 mM HEPES, 150 mM KCl, 5 mM MgCl_2_, 40 µM phenol red, pH 7.5). The reaction was initiated by the addition of 30 µM dUTP (K_M_ =3.6 ±1.9 µM^27^) after 5 min preincubation of 50 nM human dUTPase and 300 nM or varying concentration Stl variants at 25 °C. Three parallels were measured in each case. The slope of the first 10% of the progress curve was used to determine the initial velocity^28^. The quadratic binding equation was fitted to Stl variant inhibition data^21^:

1 $$\:y=s+\frac{A[\left(c+x+{K}_{\text{i},\text{a}\text{p}\text{p}}\right)-\sqrt{{\left(c+x+{K}_{\text{i},\text{a}\text{p}\text{p}}\right)}^{2}-4cx}]}{2c}$$

where *y* is the relative enzyme activity normalized to the uninhibited activity, *x* is the Stl variant concentration, *s* is the relative enzymatic activity without Stl variant, *A* is the total amplitude of the activity change, *c* is the dUTPase concentration, *K*_i, app_ is the apparent inhibitory constant.

### Biolayer interferometry

An Octet K2 system (Sartorius), with streptavidine coated SAX biosensors (Sartorius) was used to study protein-protein binding at 30 °C in a buffer containing 50 mM HEPES, 300 mM NaCl, 5 mM MgCl_2_, pH 7.5 applying at least five different dilutions. 1:1 binding model was fitted to the data using Octet Data Analysis HT program.

### Crystallization and crystallography

Stl^NT^ and hDUT proteins were gel filtrated separately in 50 mM HEPES, 300 mM NaCl, 5 mM MgCl_2_, pH 7.5 on a Superdex^®^ 200 10/300 GL column in an ÄKTA Explorer purifier system. Mixture of Stl^NT^ and hDUT in 1:1 monomer molar ratio was concentrated to ca. 20 mg/ml. Crystals were grown at 20 °C from 1:1 mixture of the concentrated protein solution with 1.5 M ammonium sulfate, 0.1 M sodium acetate pH 5 (Molecular Dimensions ProPlex F11) solution on a vapor diffusion plate with sitting drop setup. Diffraction data were collected at Elettra Synchrotron (Trieste, Italy) at beamline 11.2 C XRD2 (at wavelength of 1.000 Å). Data processing was carried out with XDS^29^ and SCALA^29,30^. The phase problem was solved by molecular replacement using PHASER of the CCP4 package^31,32^ based on Stl^NT^ and hDUT structures (PDB ID: 6H49 and 3EHW, respectively). Coot^33^ and Phenix^34^ packages were used for model building and refinement. The crystal structure is characterized by the following Ramachandran statistics: 97.28% favoured, 2.72% allowed, 0.00% outliers. Crystallographic and refinement data are summarized in Table S1. The crystal structure of the human dUTPase in complex with Stl^NT^ has been deposited to the Protein data Bank^35^ under the PDB accession code 8C8I.

### Protein structure prediction

To further investigate the potential effect of Stl mutations on Stl dimerization and complex formation with hDUT, protein structures were predicted using AlphaFold3^35^ (https://alphafoldserver.com). 5–5 models were generated in each case using the full-length Stl (UniProt: Q9F0J8) and the hDUT (UniProt: P33316-2 lacking the nuclear localisation signal 1-MPCSEETPAISPSKRARPAEVGG-23 sequence element) sequences. The designed mutations were introduced in Stl sequence and 2 copies were used as input for analysing the effect of mutations on Stl dimerization and 3 copies of Stl mutants and a hDUT trimer were given as an input in case of hDUT:Stl complex modelling.

### Protein structure comparison

Superimposition of dUTPase:Stl^NT^ crystal structures °was carried out using cealign and align commands in PyMOL 2.5.4 (Schrodinger, LLC). Per-residue Root mean square deviation (RMSD) plots were created using VMD^36^.

### Peptide inhibitor design

To test the effect of an Stl-based peptide inhibitor, a peptide was designed based on the previously identified Y98-Y113 sequence segment as a potential hDUT-interacting domain of Stl based on HDX-MS analysis^17^. In addition, the solubility of the peptide was an important factor which was estimated using PepCalc.com (Innovagen AB). The structure of the designed 95-DKMYSYVNKAYYNDGDIYYSSYD-117 peptide was predicted by PEP-FOLD 3^37^ (result shown in Supplementary Fig. S9).

### Softwares used to create figures

The illustrations of crystal structures were created using PyMOL 2.5.4 (Schrodinger, LLC), the structural superposition of crystal structures was carried out using align command in PyMOL. BLI graph and scheme were created using Octet Data Analysis software (FortéBio) and PowerPoint, respectively. Other graphs were created with OriginPro 2018 (OriginLab Corporation). Final figures from individual graphs and images were assembled using CorelDRAW 2020 (Corel Corporation).

## Results

### Structure of Stl^NT^:human dUTPase complex

**Fig. 1:**
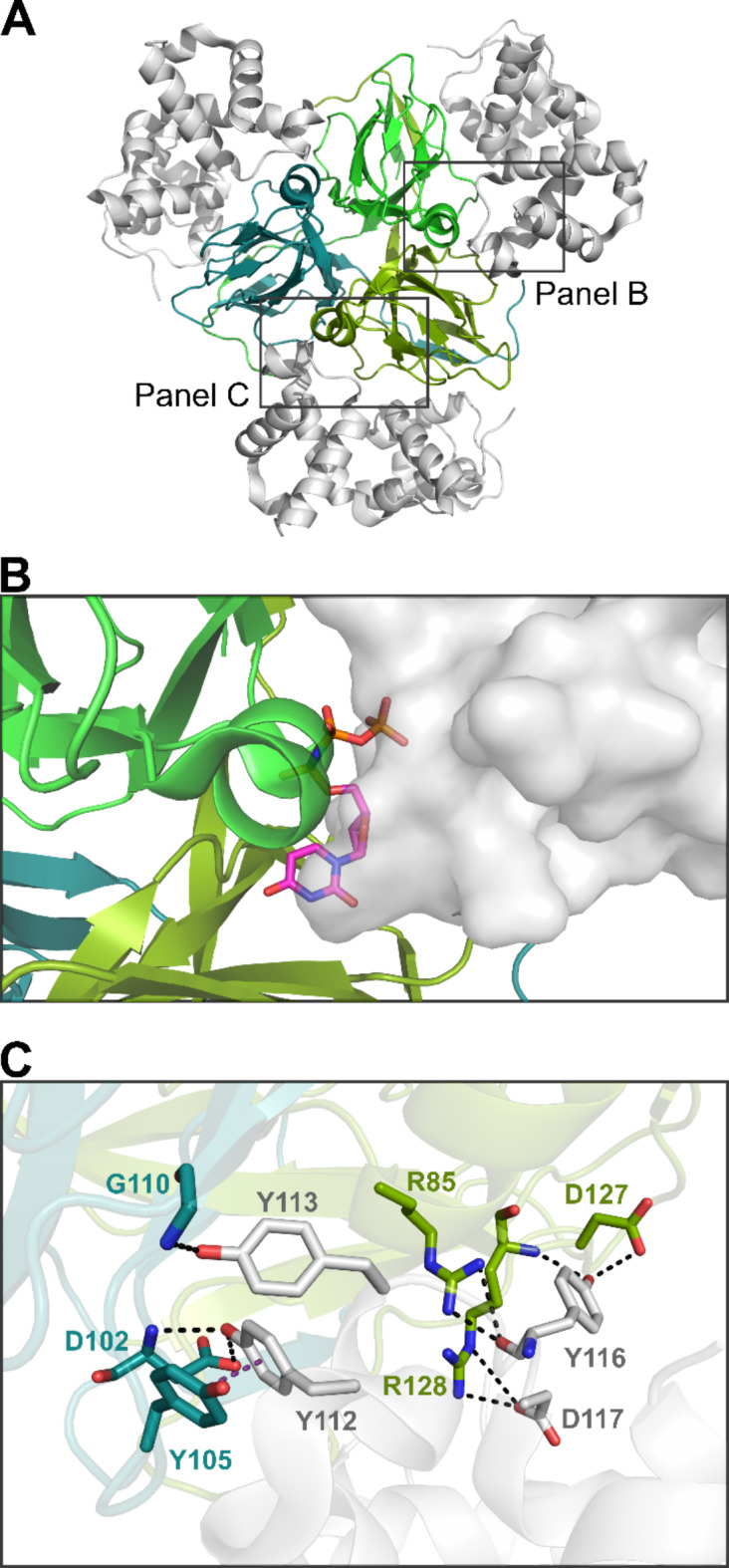
The structure of human dUTPase in complex with Stl^NT^ (PDB ID:8C8I). (**A**) Overall structure of the protein-protein complex. Human dUTPase is represented as cartoon, the three protomers are colored in different shades of green and turquoise; Stl^NT^ is shown as gray cartoon. (**B**) Interference of Stl with substrate binding. The substrate analogue dUPNPP is shown as sticks based on the structural alignment with the ligand-bound structure (PDB ID: 3EHW). The representation of human dUTPase is the same as on panel (**A**), Stl^NT^ is represented as grey surface. (**C**) Interactions within the main binding surface of Stl (tyrosine-rich Y106-D117 segment) to human dUTPase. Coloring of hDUT is according to panel (**A**). Residues forming H-bonds are shown as stick with atomic coloring (C: based on chain, N:blue, O:red), black dashed lines represent H-bond interactions, purple dashed line represent π-π stacking interaction. Individual panels were created using PyMOL 2.5.4 (Schrodinger, LLC; https://www.pymol.org/) and the figure was assembled using CorelDRAW 2020 (Corel Corporation; https://www.coreldraw.com).

We determined the structure of the human dUTPase in complex with the well folded N-terminal segment of Stl (Stl-1-159, Stl^NT^) by X-ray crystallography (PDB ID: 8C8I), to 3.2 Å resolution (Supplementary Table S1). The complex was crystallized in P2_1_2_1_2_1_ space group, and the asymmetric unit contains a trimer dUTPase bound to three Stl^NT^ chains (Fig. 1a). The overall fold of the complex is very similar to that of the φ11 phage dUTPase and the *L. vannamei* dUTPase (LvDUT) complexes with Stl^NT^ (PDB ID: 6H4C, 7DLV)^23,24^. (Supplementary Fig. S1). As proposed by our HDX-MS measurements^17^ the main binding surface of Stl within the crystallized complex was the tyrosine-rich Y106-D117 segment, which interacts with residues of the human dUTPase active site (conserved motifs 2,3,4) (Fig. 1b, c). In case of the phage DUT-Stl^NT^ complex the N-terminal of the dUTPase (residues: R15, E18, N20, H21, D24) serves as a secondary interaction surface with Stl (residues D77, R74, Y70 and Y98, N102, Y106 respectively). These interactions are missing in case of hDUT and LvDUT, as the residues in the matching positions are unable to interact with Stl (Supplementary Fig. S2). This is consistent with the weaker binding of hDUT and LvDUT to Stl as compared to φ11 phage dUTPase reported previously^24^. In concordance with the stronger DUT-Stl interaction the φ11 phage dUTPase is fully inhibited by Stl while the human and shrimp dUTPases retain significant activity (30% and 35% respectively) even in the presence of excess Stl. During this work, another crystal structure of human dUTPase in complex with Stl^NT^ has been published (PDB ID: 7PWJ), which also reinforces our findings^38^ (Supplementary Fig. S3). The two complex structures share a relatively high degree of similarity based on root mean square deviation (RMSD) of 1.5 Å for 527 Cα atoms (Supplementary Fig. S3a). Based on per-residue RMSD values, the hDUT protomers are very similar in these structures, except for a few residues at the N-terminal and C-terminal segments of the dUTPase chains (Supplementary Fig. S3b). In addition to our results the C-terminal E152-G159 segment hDUT is resolved in the 7PWJ structure which interacts with the G66-P68 and the Y98-N102 regions of Stl^NT^. The Stl^NT^ chains from the two crystal structures show less similarity (Supplementary Fig. S3c, d), suggesting their greater flexibility in the complex. Apart from the Stl^NT^ chain ends, the highest degree of distortion is observable on the G37-V55 segment of one of the chain pairs, associated with the highest B-factor values, which includes the region between the end of helix α2 and the beginning of helix α4 (Supplementary Fig. S3c–e). This distortion in the other Stl^NT^ chain pairs applies only to the loop connecting helices α3 and α4. Additional short flexible regions are present in E81-Y84 and K130-K138 Stl^NT^ segments (Supplementary Fig. S3c, d,f, g). The residues of Stl which interact with hDUT are positioned very similarly in the two structures.

### Structure based design of Stl point mutants with potentially enhanced human dUTPase inhibition ability

**Fig. 2:**
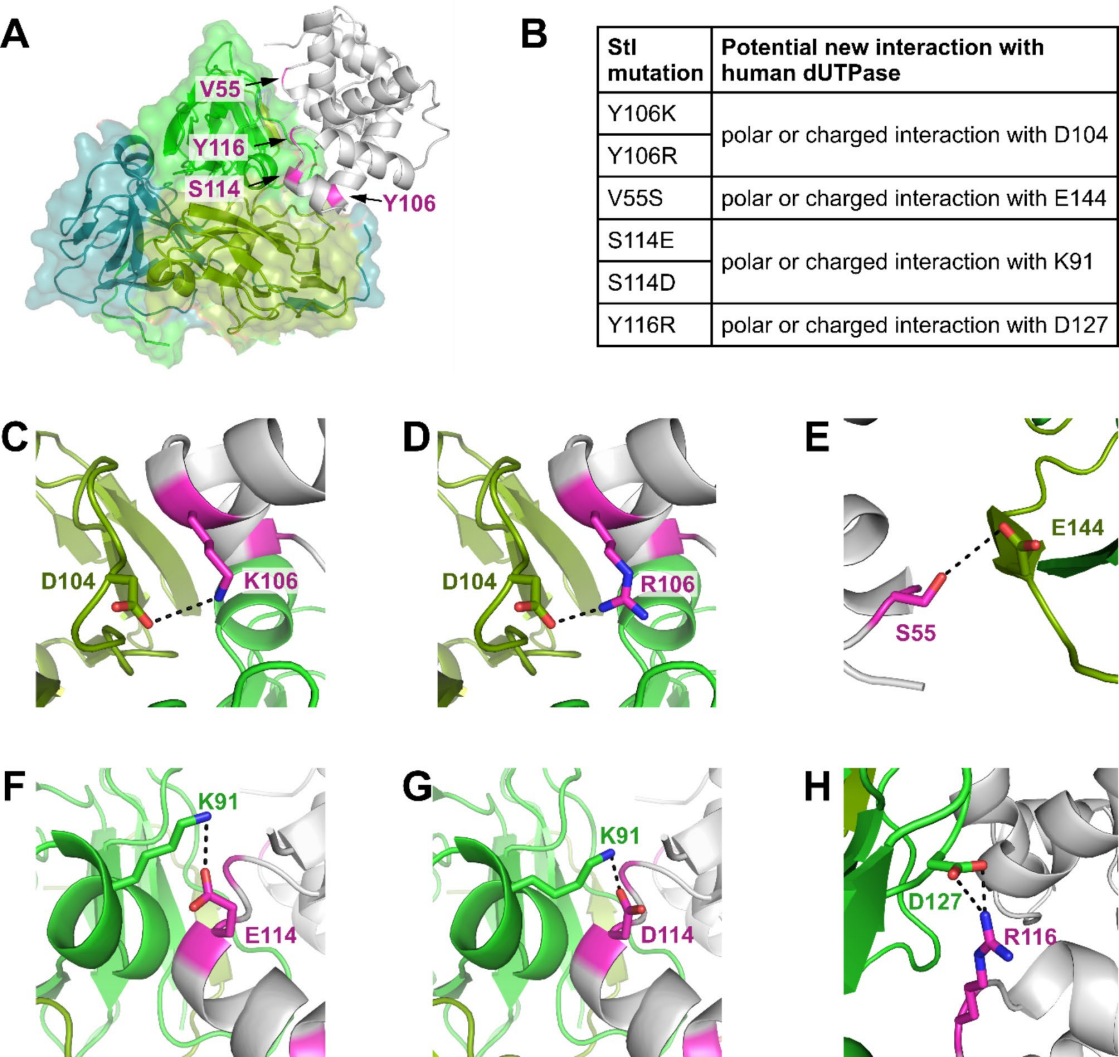
Stl point mutant constructs aiming to increase its interaction with the human dUTPase (**A**) Spatial position of the mutations. hDUT is represented as cartoon and partially transparent surface colored as on Fig. 1, Stl represented as grey cartoon, mutation positions are highlighted in magenta. (**B**) List of expected new interactions. (**C**–**H**) 3D representation of the predicted newly formed H-bonds. hDUT representation is the same as on Fig. 1, interacting residues are shown as sticks, Stl is grey, mutated residue is shown as sticks with atomic coloring (C: magenta, N: blue, O: red). Individual panels (**A**,**C**–**H**) were created using PyMOL 2.5.4 (Schrodinger, LLC; https://www.pymol.org/), panel (**B**) was created and the figure was assembled using CorelDRAW 2020 (Corel Corporation; https://www.coreldraw.com).

To enhance the inhibitory effect of Stl on hDUT we designed point mutant Stl constructs based on our crystal structure targeting the main interaction surface of hDUT-Stl^NT^ complex, aiming to increase the interaction between the two proteins (Fig. 2).

In case of φ11 phage dUTPase sidechain of Stl-Y106 forms H-bond interactions with the N-terminal of the enzyme (residues H21, D24), while it has no detectable interaction with hDUT. We hypothesized that replacement of tyrosine to a residue with positively charged side chain may result in interaction with D104 of motif 3 of human dUTPase.

Besides this the replacement of Stl-S114 with residues of negatively charged side chain can potentially lead to interaction with K91 of the human dUTPase. We also wished to test the effect of Y116 to arginine, to enhance the interaction with D127 of hDUT.

Based on the crystal structure of Stl^NT^, residues to be mutated (i.e. V55, Y106, S114, Y116) are found on the surface of the protein forming no sidechain interactions with the core of the protein, with the exception of the sidechain of S114, which interacts with the mainchain of D110 (Supplementary Fig. S4). Thus, no major effect of the planned mutations on the integrity of Stl was expected. In order to examine whether these mutations may result in formation of polar interactions in the context of hDUT-Stl complex as we predicted, we have analyzed the possible interactions between the targeted residues using AlphaFold modelling (Supplementary Fig. S5). Most of the models show the formation of very similar interactions as presented in Fig. 2, while the mutations targeting Stl S114 residue (S114E, S114D) resulted in formation of H-bond between side chain of hDUT K91 residue and the main chain of the mutated Stl residue.

We tested the stability of the mutants by thermofluorimetry (cf. Table 1). Most of the mutants showed similar stability as the wild-type Stl (T_M_ = 54.3 °C), except S114D and S114E mutants which showed reduced thermal stability (T_M_ = 47.8 °C and T_M_ = 49.5 °C respectively). To further investigate the impact of these mutations, we used AlphaFold modeling to assess whether these influence the dimer formation of the Stl protein. The obtained models imply that similarly to Stl^WT^, the homodimerization surface is formed between the C-terminal domains and the conformation of N-terminal domains are considerably similar to that of Stl^WT^ (Cα-RMSD values less than 0.54 Å in case of all Stl mutants). The relative orientation of the N-terminal domains to the C-terminal regions was found to be highly variable within the top 5 models for Stl^WT^ and also in the case of the six mutant Stl proteins (Supplementary Fig. S6a-j). This is in accordance with our earlier SEC-SAXS measurements of Stl^WT^, which revealed inherent inter-domain flexibility of the Stl protein^17^, which likely remained unaltered by the introduction of the mutations.

Enzyme activity measurements revealed that none of the six mutants showed better inhibitory potential compared to the wild-type Stl protein (Table 1). Based on the models we expect no major change in the structure of these mutant Stl proteins and their complexes with hDUT (cf. Supplementary Fig. S5 and S6). We suggest that the flexible polar side chains of the newly introduced residues (aspartate, glutamate, arginine, lysine), that can be stable in multiple conformations adopted different orientation than expected from the predictions. It seems that the most favorable conformation of the mutated Stl residues did not result in the formation of a new H-bond with the human dUTPase. Thus, we turned to another approach to design a more potent human dUTPase inhibitor from the Stl protein.

**Table 1:** Thermal stability of Stl mutants and their inhibitory potential on the human dUTPase.

	Tm*	Residual activity of 50 nM hDUT at 300 nM Stl concentration
Stl-WT	54.3 ± 0.24	30%
DUT: Stl interaction surface mutants		
Stl-Y106R	55.0 ± 0.0	89%
Stl-Y106K	54.2 ± 0.1	83%
Stl-S114D	47.8 ± 1.1	100%
Stl-S114E	49.5 ± 0.3	100%
Stl-Y116R	58.5 ± 0.0	69%
Stl-V55S	53.0 ± 0.0	75%
Dimer interface mutants		
Stl-E189A	52.5 ± 0.0	77%
Stl-I181A, I184A	49.0 ± 0.0	70%
Stl-L192A, F196A	53.7 ± 0.5	100%
Truncated derivatives		
Stl^NT^ (residues 1-159)	55.5 ± 0.0	50%
peptide (residues 95–117)	NA	70%

*Average and standard deviation of three parallel measurements.

### Design of Stl dimer interface mutants with potentially enhanced human dUTPase inhibition ability

Our recent biolayer interferometry measurements directly proved the previous hypothesis drawn from SEC-SAXS that Stl dimerization interferes with its binding and inhibition of the human dUTPase^26^. Thus, we hypothesized that mutations on the dimer interface perturbing the oligomerization of Stl could enhance its inhibitory effect exerted on the human dUTPase.

**Fig. 3:**
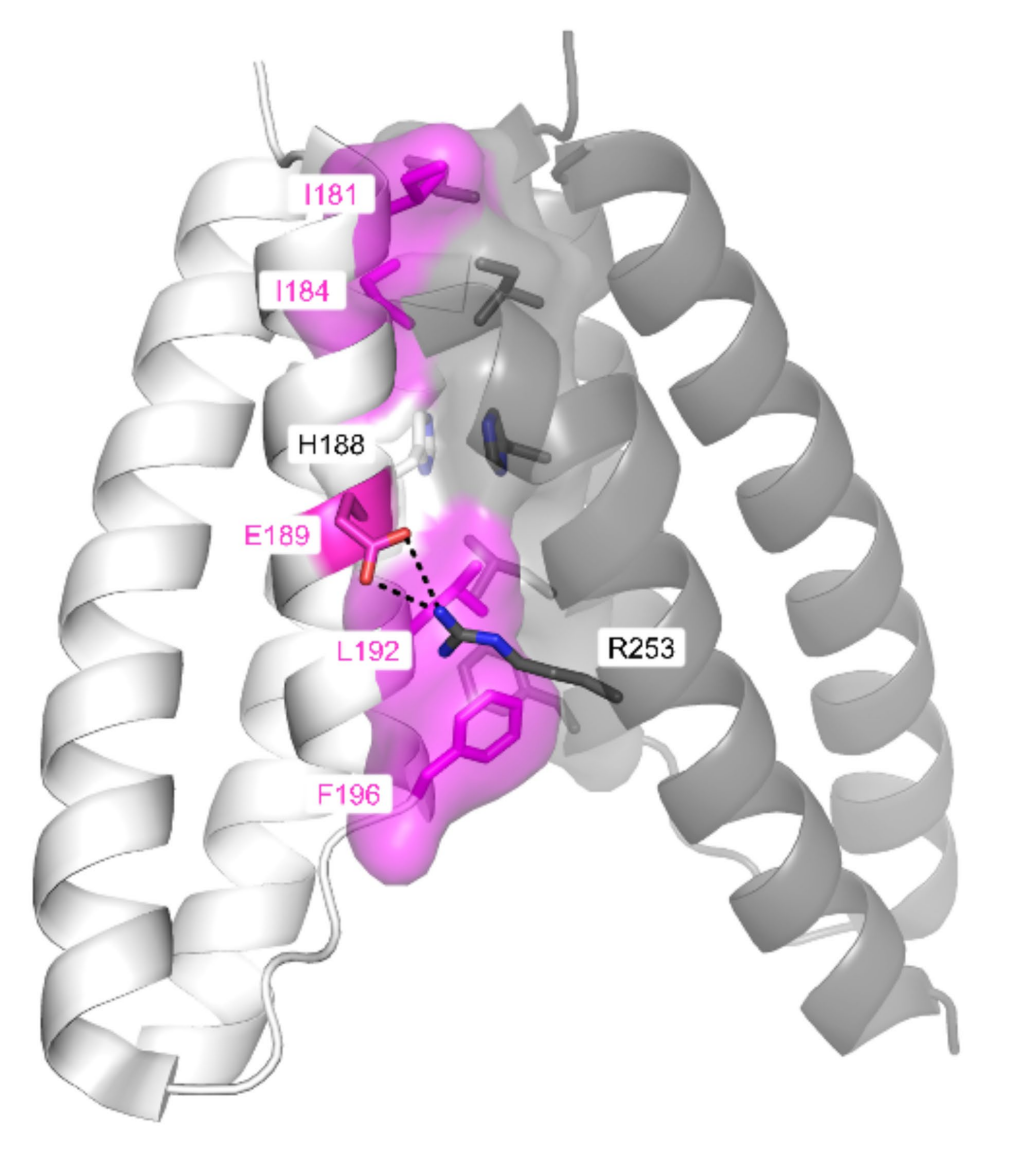
Dimer interface of Stl (Stl C-terminal segment residues 175–267, PDB ID: 6H48). The two chains of the dimer are shown as white and gray cartoons. Residues on the dimer interface are shown as sticks, surface of residues that are involved in van der Waals interactions are shown, black dashed line represents the H-bond interaction between the two chains of the dimer. Mutated residues are colored with magenta on one chain of the dimer. Figure was created using PyMOL 2.5.4 (Schrodinger, LLC; https://www.pymol.org).

We mutated dimer interface residues to alanine (Fig. 3), and produced three Stl constructs (Stl-E189A, Stl-I181A, I184A, Stl-L192A, F196A) carrying single and double point mutations.

Based on the observed thermal stability of these three mutant proteins (Table 1), only the double mutant Stl-I181A, I184A caused significant destabilization of the dimeric protein. In addition to the thermal stability analysis to investigate whether these mutations affected the homodimerization of Stl C-terminal segment we generated AlphaFold models (Supplementary Fig S6h–j). These models show that the dimerization of Stl was perturbed as the C-terminal regions are positioned apart in space.

Enzyme activity measurements indicated that these mutations at the C-terminal segment of Stl aiming to disrupt only the dimerization of Stl also impaired the inhibitory potential of the protein on the human dUTPase (Table 1). We measured the inhibitory curve of Stl-I181A, I184A, which was the best performing dimer interface Stl mutant, and found that the maximal inhibition efficiency was approximately 30%, and the determined apparent inhibitory constant (K_i, app_ ) was (2.3 ± 1.8) nM (Supplementary Figure S7c). Therefore the binding strength of Stl-I181A, I184A to hDUT in presence of dUTP substrate is similar to that of the wild type Stl, however the attempt to disrupt the homodimerization of Stl with these mutations resulted in a less potent inhibitor. Therefore, we conclude that the C-terminal segment of Stl contributes to the inhibition of the human dUTPase.

### Inhibition of human dUTPase by truncated derivatives of Stl inhibitor protein

**Fig. 4:**
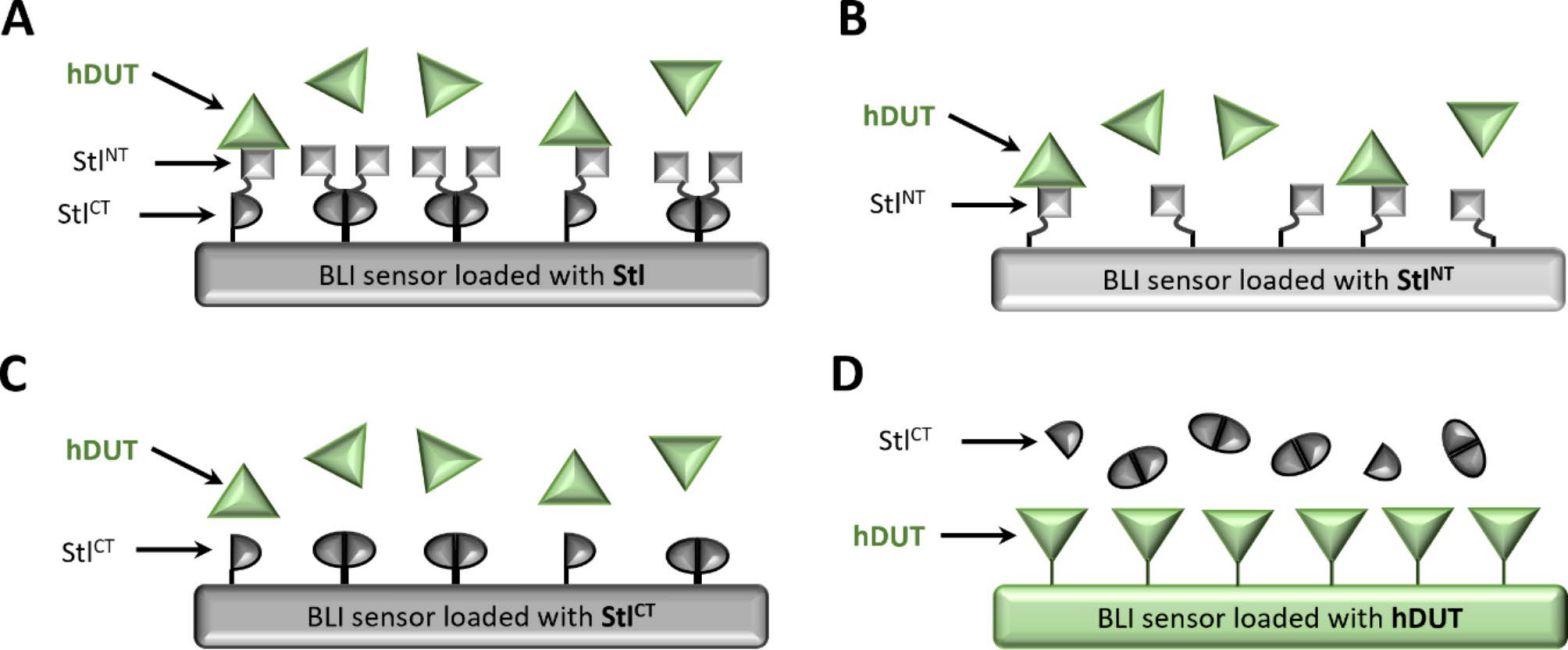
Schematic figure showing experimental setups of BLI measurements, data obtained are listed in Table 2. Figure was created using Power Point (Microsoft Corporation).

As our efforts with the perturbation of dimerization of the full-length Stl did not lead to an enhanced dUTPase inhibitor we wished to test how removal of the full C-terminal dimerization domain affects the ability of Stl to inhibit the human dUTPase. Hence, we tested the inhibitory effect of the well folded N-terminal segment of Stl (Stl-1-159, Stl^NT^), used for X-ray crystallographic structure determination of Stl-dUTPase complexes, on human dUTPase. The maximal inhibitory effect of Stl^NT^ protein was found to be only ca. 50% (cf. Supplementary Fig. S7a), while the apparent inhibitory constant of Stl^NT^ on hDUT, was larger (K_i, app_ =31 ± 6 nM) than that of the full-length Stl protein (K_i, app_ =7 ± 2 nM)^17^. To test if this effect is due to weaker binding of Stl^NT^ compared to that of full-length Stl protein we conducted biolayer interferometry (BLI) experiments, which allows us to analyze the binding of the two proteins in a substrate-free environment. We found that the binding kinetics and strength were similar for the truncated Stl^NT^ (Supplemetary Fig. S8) and the full-length Stl protein (Table 2). It is important to note here that we attached Stl and Stl^NT^ to the sensor (Fig. 4a, b). This method of choice is beneficial since it eliminates the effect of dimerization of full-length Stl and multiple binding of either Stl variants to the human dUTPase trimer^26^. The observed difference between the K_D_ values obtained by BLI and the apparent inhibitory constants (K_i, app_) could be attributed to the competitive binding between the Stl variants and the substrate during the measurement of the latter.

We suggest that the observed residual activity is caused by the combination of relatively slow inhibitor binding and the high substrate concentration. The excess substrate competes effectively for the active site, and despite the high affinity of the inhibitor, its slow binding prevents Stl from efficiently blocking dUTP binding and fast catalysis. As a result, some enzymatic turnover occurs, leading to residual activity even with excess inhibitor and pre-incubation. While allosteric mixed inhibition could also account for the observed inhibition curve, our HDX-MS studies of the Stl-human dUTPase binding surface did not reveal any unambigous secondary binding sites for Stl^17^, and thus, based on the experimental data we consider the kinetic-based explanation to be more plausible.

We also tested the binding of Stl^CT^ (residues 175–267) to the human dUTPase and observed no detectable binding of the two proteins to each other either if Stl^CT^ or dUTPase was bound to the sensor (Fig. 4c, d). We concluded that the C-terminal part of Stl does not contribute to the binding of the inhibitor to the human dUTPase but has an important role in the inhibition of the enzymatic activity in this specific case.

**Table 2:** Biolayer interferometry data on human dUTPase interaction with Stl, Stl^NT^ and Stl^CT^. Experimental setups are shown on Fig. 4.

	Figure 4	K_D_ (nM)	k_a_ (µM^− 1^ s^− 1^)	k_dis_ (10^− 4^ *1/s)	χ^2^
Stl + hDUT ^26^	Panel A	0.179 ±0.001	1.53±0.00	2.73·± 0.03	1.019
Stl^NT^ + hDUT	Panel B	0.177± 0.002	0.60±0.00	1.06 ± 0.01	0.427
Stl^CT^ + hDUT	Panel C	No binding			
hDUT + Stl^CT^	Panel D	No binding			

Based on HDX-MS and 3D crystal structures a peptide segment of Stl including residues 106–113 directly binds to the active site of the enzyme. Thus, we tested effect of a peptide 95-DKMYSYVNKAYYNDGDIYYSSYD-117 on the human dUTPase enzymatic activity and found that even at eight times molar excess the maximal inhibitory effect is 30% (Supplementary Fig. S7), which is significantly less than the 70% inhibition of the full-length Stl protein^17^, although the apparent inhibitory constant of the peptide on the human dUTPase (K_i, app_ = 13 ± 4 nM) was in the same range as that of Stl and Stl^NT^ proteins.

To find a possible explanation for the lower inhibitory efficiency of the peptide, we predicted its structure on its own and in complex with human dUTPase. The results suggest that the peptide may adapt similar but more flexible conformation compared to its embedded segment in Stl^NT^ interacting with hDUT (Supplementary Fig S9a, b). The hDUT-peptide complex model shows the peptide in a more opened conformation and its 112-YYSSY-116 segment is bound into the active site of hDUT interfering with substrate binding (Supplementary Fig. S9c, d). However, the C-terminal arm of the enzyme comprising conserved motif 5 is not displaced by the peptide probably due to its small size, which may result in a less potent competition between the dUTP substrate and the peptide in active site-binding.

We assume that this lesser inhibitory effect might be also due to greater flexibility of the peptide, as this segment of residues 95–117 (including helices α7 and α8) has some interactions with the other part of the Stl protein (with helices α5, α6, α9 and α10) within the complex, thus it might be less capable to fold to the ample conformation for binding and inhibition.

## Discussion

Since the active site of trimeric dUTPases is built up by well-conserved motifs throughout the various classes of life the proteinaceous inhibitor of the φ11 phage dUTPase was found to be effective on several other dUTPases, although the extent of inhibition varies between the proteins from different species^17,19,20,24^. It has also been demonstrated earlier that this inhibitory effect of Stl on trimeric dUTPases is not entirely universal, as the wild-type *E. coli* dUTPase although able to bind Stl, its activity is not perturbed by that^21^. It was shown that mutation of a key residue on the surface of *E. coli* dUTPase resulted in a modified enzyme that is inhibited by Stl. Thus, we hypothesized that it is also possible to design mutations on the surface of Stl leading to enhanced inhibitory effect on the human dUTPase, compared to that of the wild-type Stl, with which 30% residual dUTPase activity is retained. Based on the crystal structure of the human dUTPase with the amino terminal domain of Stl presented in this work (PDB ID: 8C8I), we designed six different point mutations on Stl aiming to form new hydrogen bonds with the human dUTPase. This rational mutagenesis approach did not lead to better hDUT inhibitor variants, likely due to the flexibility of the introduced polar sidechains the predicted H-bonds may not have been formed.

Based on HDX-MS and X-ray crystallography data a short segment consisting of residues 95–117 of Stl is responsible for binding of the inhibitor protein to the active site of various trimeric dUTPases^17,18,23,24,38^. Here we demonstrated that this motif on its own and if displayed on C-terminally truncated Stl (residues 1-159, Stl^NT^) has less inhibitory effect on human dTPase activity than the full-length Stl protein also shown in case of *M. tuberculosis* dUTPase-Stl variant interaction^39^. The decreased inhibitory capacity of the peptide 95–117 may be, at least partially, due to the fact that the peptide on its own is expected to exhibit an increased degree of conformational flexibility as compared to the situation when it is embedded in the Stl structure.

We showed that the decrease of inhibition in case of Stl^NT^ is not due to weaker binding as the equilibrium binding constant by BLI and the apparent K_i_ were similar in case of the full-length Stl and the Stl^NT^ constructs. As the direct binding of the Stl^CT^ protein (consisting of residues 175–267) to human dUTPase was not detectable, the role of this segment in the inhibition is rather due to steric effects.

The dUTPase enzymatic reaction imply complex conformation changes of the flexible fifth conserved motif at the C-terminal of the enzyme^27,40^. This motif 5 closes the active site and fixes the phosphate chain of the substrate dUTP in a conformation that is favorable for hydrolysis.

We suggest that the flexibility of dUTPase motif 5, the binding strength and the steric hindrance ability of the inhibitory protein together determine the extent of inhibition exerted by an Stl variant on a specific dUTPase enzyme. Thus, we concluded that the inhibition is a result of a delicate interplay between the two proteins. Hence, a more high-throughput, directed evolution or artificial intelligence based methods would perhaps been needed to find Stl mutants with enhanced inhibitor potency for a specific dUTPase.

## Electronic supplementary material

Below is the link to the electronic supplementary material.

Supplementary Material 1

## Data Availability

Protein Data Bank accession number: The crystal structure of the human dUTPase in complex with Stl (a potent proteinaceous inhibitor) has been deposited to the Protein data Bank (www.pdb.org) under the accession number PDB ID 8C8I. The data supporting the findings of this study are available within the paper and its Supplementary Information.
