# Supplementary material for: Full-length inhibitor protein is the most effective to perturb human dUTPase activity
Source: Sci Rep. 2025 Feb 9;15:4836. doi: 10.1038/s41598-025-86131-7 (PMC11808092; doi:10.1038/s41598-025-86131-7)
Supplement: Supplementary file 1 — Supplementary Material 1 [file 41598_2025_86131_MOESM1_ESM.pdf]

# SUPPORTING INFORMATION

# Full-length inhibitor protein is the most effective to perturb human dUTPase activity

**Bianka Kőhegyi[1,2,#], Zoé S. Tóth[2,3,#], Enikő Gál[1,2], Máté Laczkovich[1,2], András Benedek[1,2], Beáta G. Vértessy[1,2,*], Kinga Nyíri[1,2,*]**

[1] Department of Applied Biotechnology and Food Science, Faculty of Chemical Technology and Biotechnology, Budapest University of Technology and Economics, Műegyetem rkp. 3., H-1111 Budapest, Hungary

[2] Institute of Molecular Life Sciences, HUN-REN Research Centre for Natural Sciences, Magyar tudósok krt 2. H-1117 Budapest, Hungary

[3] Doctoral School of Biology, Institute of Biology, ELTE Eötvös Loránd University, Pázmány Péter sétány 1/A, H-1117 Budapest, Hungary

[*] To whom correspondence should be addressed. Email: nyiri.kinga@vbk.bme.hu, vertessy.beata@ttk.hu

[#] These authors contributed equally to the work.

## Supplemental figures

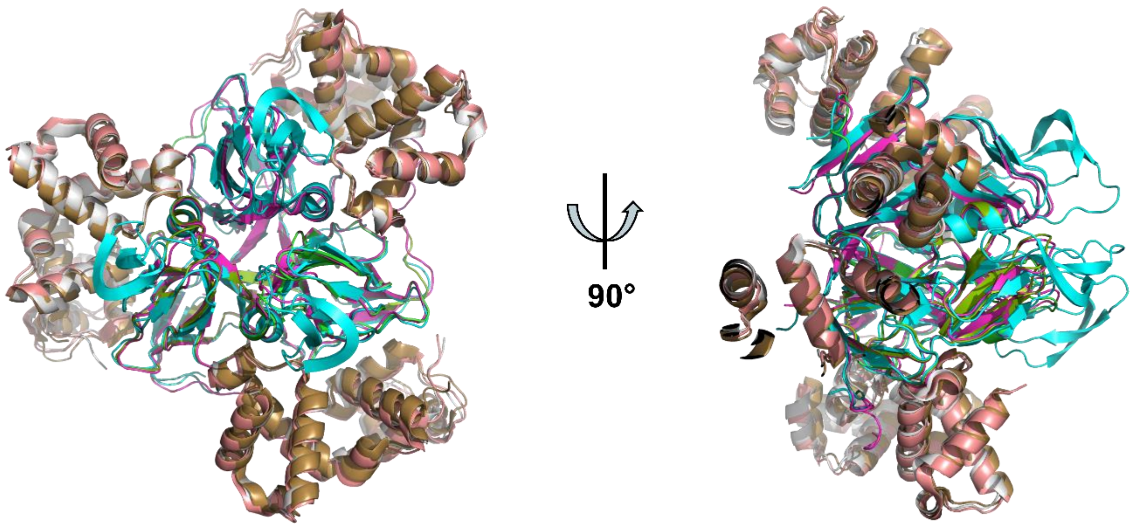


**Figure S1. Superimposition of human dUTPase: Stl$^{NT}$ complex (PDB ID:8C8I) with ϕ11 phage dUTPase: Stl$^{NT}$ (PDB ID:6H4C) and *L. vannamei* dUTPase: Stl$^{NT}$ complexes (PDB ID: 7DLV).** dUTPases are represented as green (human), cyan (ϕ11 phage) and magenta (*L. vannamei*) cartoons. Stl$^{NT}$ molecules are shown as grey (human), light pink (ϕ11 phage) and brown (*L. vannamei*) cartoons. Structural model images were created with PyMOL 2.5.4 (Schrodinger, LLC; https://www.pymol.org)

and the figure was assembled using CorelDRAW 2020 (Corel Corporation; https://www.coreldraw.com).

```
                                            Motif 1
hDUT     (24)       --MQLRFARLSEHATAPTRGSARAAGYDLYSAYDYTIPPMEKAVVKTDIQIALPSGCYGR
LvDUT    (68)       LKSVLRFKKLTEHAFTPSKGSKFAAGFDLCSAYDLVIPAVGKALVKTDIQVELPEGCYGR
ϕ11DUT    (1)       MTNTLQVRLLSENARMPERNHKTDAGYDIFSAETVVLEPQEKAVIKTDVAVSIPEGYVGL
                        *:.  *:*:*  * :.    **:*: **   .: .  **::***: : :*.*  *

                     Motif 2                Motif 3
hDUT     (82)       VAPRSGLAAKHFIDVGAGVIDEDYRGNVGVVLFNFGKEK---------------------
LvDUT   (128)       IAPRSGLSWKHHIDVGAGVIDRDYRGNVGVVLFNHAKTD---------------------
ϕ11DUT   (61)       LTSRSGVSSKTHLVIETGKIDAGYHGNLGINIKNDAIASNGYITPGVFDIKGEIDLSDAI
                    ::.***:: * .: : :* ** .*:**:*: : * .  .

                              Motif 4                          Motif 5
hDUT    (121)       -----FEVKKGDRIAQLICERIFYPEIEEVQALDDT-ERGSGGFGSTGKN
LvDUT   (167)       -----YEVKKGDRVAQLICEKIIYPEIQEVEELMET-ERGEGGFGSTGNQ
ϕ11DUT  (121)       RQYGTYQINEGDKLAQLVIVPIWTPELKQVEEFESVSERGEKGFGSSGV-
                         :::::**::***:   *  **:::*: : .. ***. ****:*
```

**Figure S2 Sequence alignment of human, shrimp and ϕ11 dUTPase proteins.** Conserved motifs are bold, residues interacting with Stl are highlighted with yellow, residues of hDUT targeted by Stl mutations are highlighted with magenta. (Identical residues denoted with *; strongly similar residues denoted with :, similar residues denoted with .). Figure was created using Microsoft Office 365 (Microsoft Corporation).

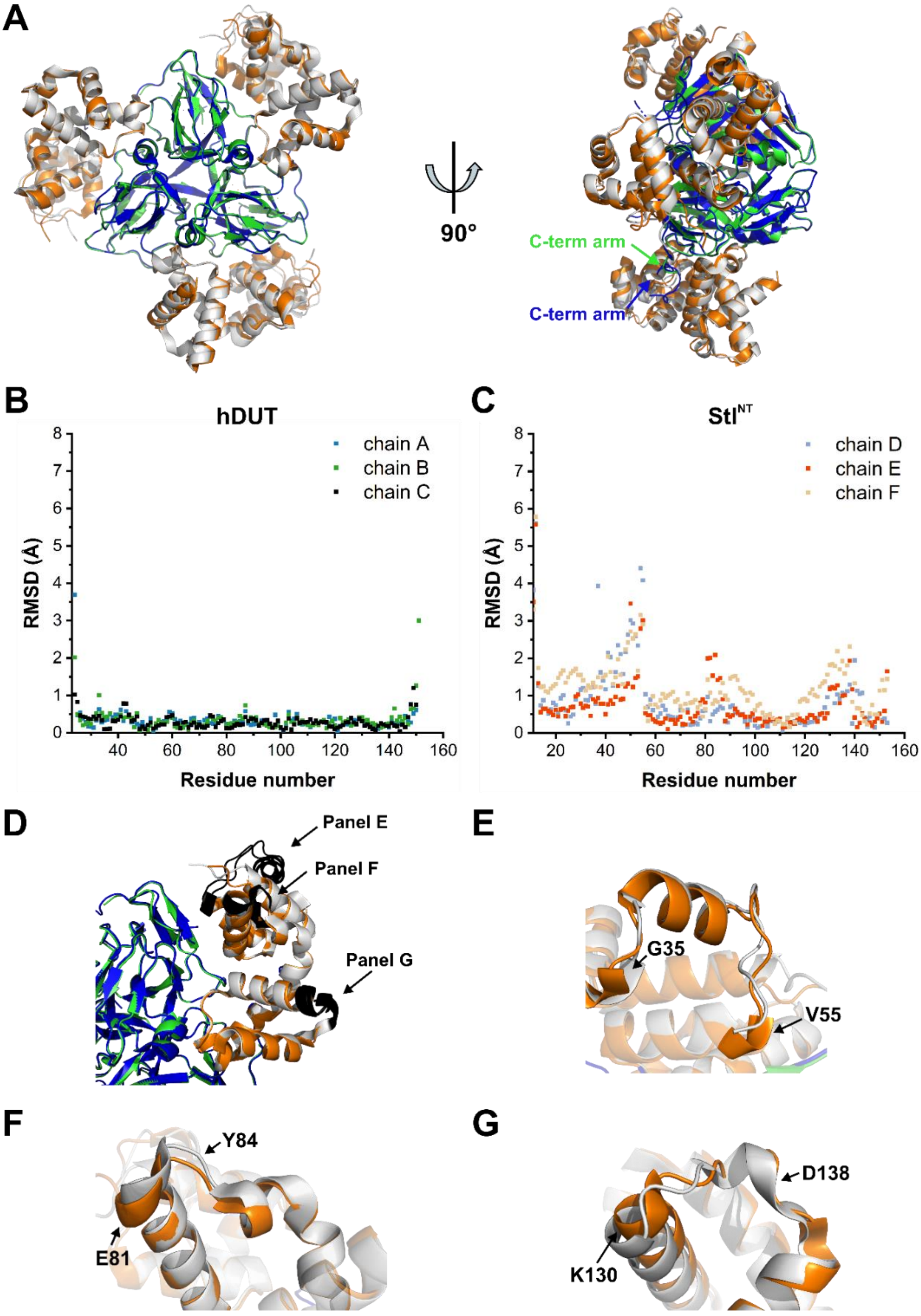


**Figure S3. Structural comparison of human dUTPase: Stl$^{NT}$ complexes (PDB ID:8C8I and7PWJ).** A) Superimposition of complex structures. hDUT trimers are displayed as green (8C8I) and dark blue (7PWJ) cartoons, Stl$^{NT}$ molecules are represented as grey (8C8I) and orange (7PWJ) cartoons. The superimposed structures are displayed at two orientations. Green and blue arrows are indicating the difference in length and conformation of hDUT C-terminal arm regions. B) Root mean square deviation (RMSD) of Cα atoms obtained from superimposition of hDUT chains (Chains A, B, C of 8C8I with the respective chains of 7PWJ). C) Root mean square deviation (RMSD) of Cα atoms obtained from superimposition of Stl$^{NT}$ chains (Chains D, E, F of 8C8I with the respective chains of 7PWJ).

D) Representation of the positions of three regions of Stl$^{NT}$ showing high RMSD values. The hDUT-Stl$^{NT}$ complexes are displayed similarly as on Panel A, the three flexible Stl$^{NT}$ regions are highlighted in black. E-G) Close up of the of three regions of Stl$^{NT}$ the most substantial difference between the Stl$^{NT}$ chains within the two experimental structures. E) Representation of Stl$^{NT}$ G37-V55 segment of chain D of 8C8I (grey) and the respective chain of 7PWJ (orange). F) Stl$^{NT}$ E81-Y84 segment of chain E of 8C8I (grey) and the respective chain of 7PWJ (orange). G) Stl$^{NT}$ K130-D138 segment of chain F of 8C8I (grey) and the respective chain of 7PWJ (orange). The beginning and the end of these segments on Panels E-G are indicated by arrows and the abbreviation of the residues. Individual panels (A, D, E. F, G) were created using PyMOL 2.5.4 (Schrodinger, LLC; https://www.pymol.org/), panels (B, C) were created with with OriginPro 2018 (OriginLab Corporation) and the figure was assembled using CorelDRAW 2020 (Corel Corporation; https://www.coreldraw.com).

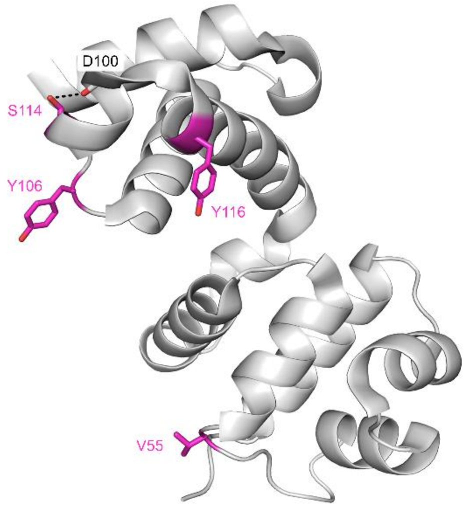


**Figure S4. Positions of mutations on Stl$^{NT}$ (PDB ID:6H49).** Protein is shown as grey cartoon, mutated residues are shown as sticks with atomic coloring (C: magenta/grey, O:red), H-bond represented as black dashed line. The figure was created using PyMOL 2.5.4 (Schrodinger, LLC; https://www.pymol.org/).

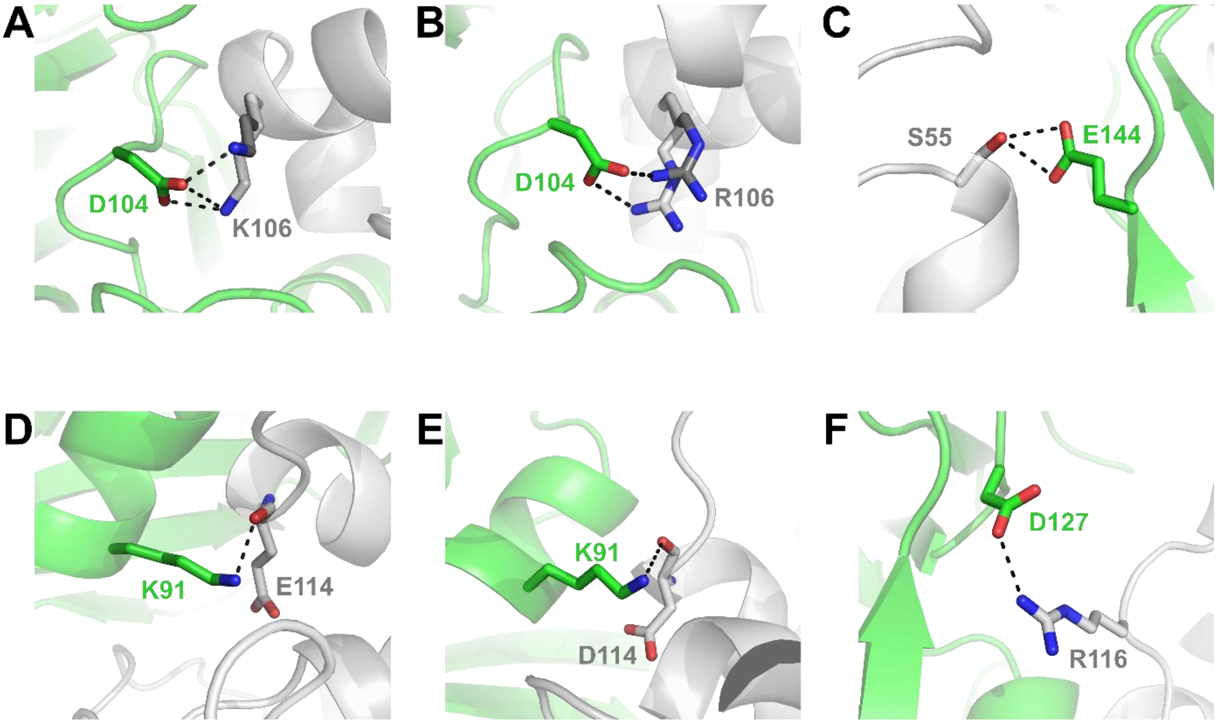


**Figure S5. AlphaFold models showing potential polar interactions between targeted main interaction surface residues in complex of hDUT and Stl mutants.** Potential polar interactions between hDUT D104 and Stl Y106K (Panel A), hDUT D104 and Y106R (Panel B), hDUT E144 and Stl V55S (Panel C), hDUT K91 and Stl S114E (Panel D), hDUT K91 and Stl S114D (Panel E), hDUT D127 and Stl Y116R (Panel F) mutated residues. The hDUT is represented as green cartoons, interacting residues are shown as sticks and colored as the cartoon. The mutated Stl proteins are represented as grey cartoons, the interacting residues are shown as sticks and colored the same as the cartoons. In case of Panels A and B alternative conformations of the mutated Stl residues (light and dark grey) and their interactions with the targeted hDUT residue is shown. Individual panels were created using PyMOL 2.5.4 (Schrodinger, LLC; https://www.pymol.org/) and the figure was assembled using CorelDRAW 2020 (Corel Corporation; https://www.coreldraw.com).

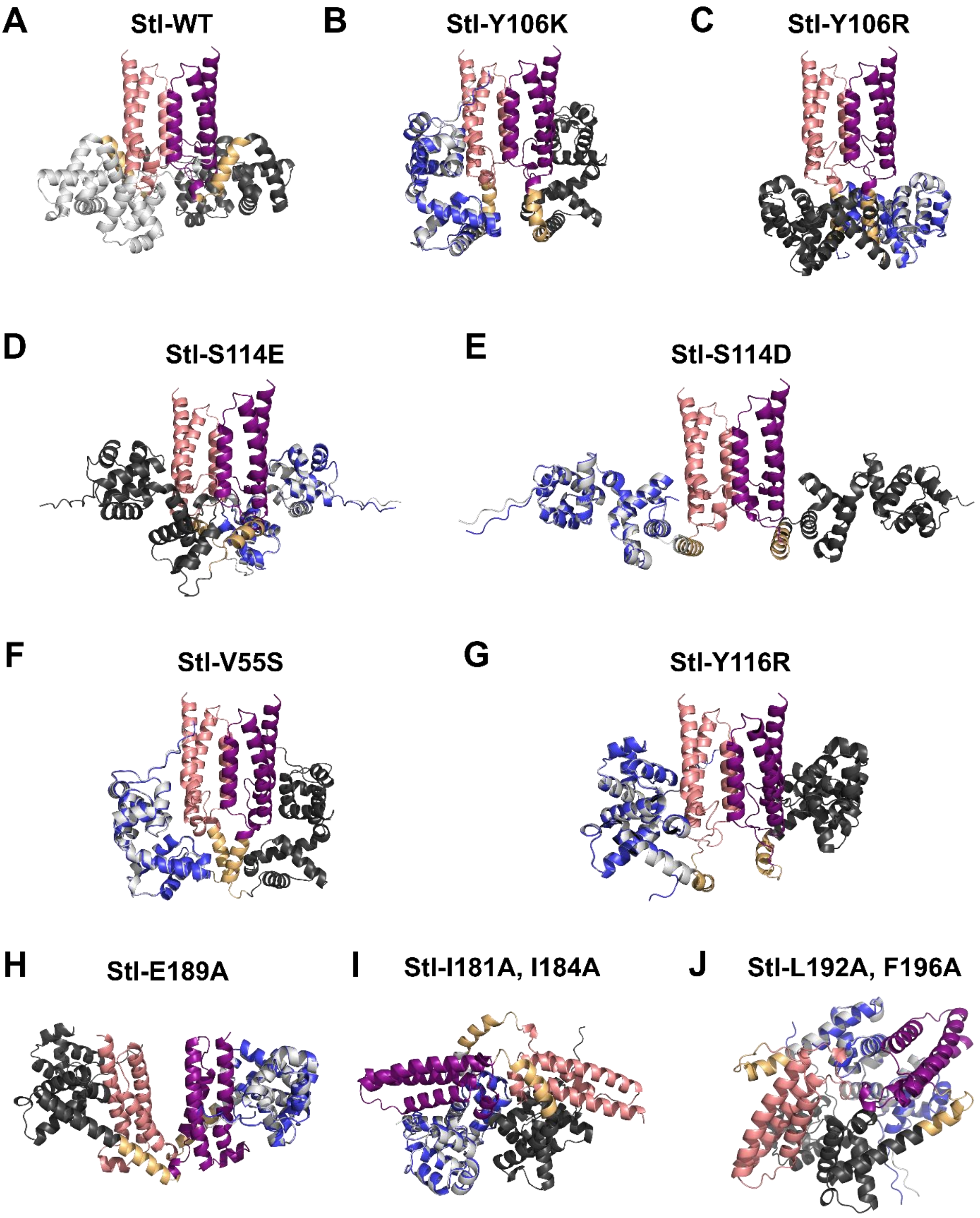


**Figure S6. Representative AlphaFold models showing potential effect of the introduced mutations on the dimerization of Stl.** A) Model showing one possible orientation of N-terminal and C-terminal regions of the wild type Stl protein ($Stl^{WT}$) in its dimeric form. The N-terminal regions ($Stl^{1-159}$) are shown as grey and black cartoons, the C-terminal regions ($Stl^{175-267}$) are shown as light pink and dark purple cartoons and the linker segments connecting these regions are shown as yellow cartoon. B-G) Models representing possible dimer architecture of Stl mutants targeting the main hDUT-interacting surface. The representation of Stl regions is similar as on Panel A. The modelled N-terminal region of

$Stl^{WT}$ aligned to one of the corresponding mutant $Stl^{NT}$ regions is displayed as blue cartoon showing high degree of conformational similarity in each case. The designation of Stl mutations is indicated above each models. H-J) Models representing the disruption of dimerization of Stl C-terminal regions. (cf. The light pink and dark purple cartoons, representing the C-terminal segments, are spatially separated from each other). The representation of Stl regions is similar as on Panel A. The display of aligned $Stl^{NT}$ region of $Stl^{WT}$ is similar to that on Panels B-G. The designation of Stl mutations is indicated above each model. Individual panels were created using PyMOL 2.5.4 (Schrodinger, LLC; https://www.pymol.org/) and the figure was assembled using CorelDRAW 2020 (Corel Corporation; https://www.coreldraw.com).

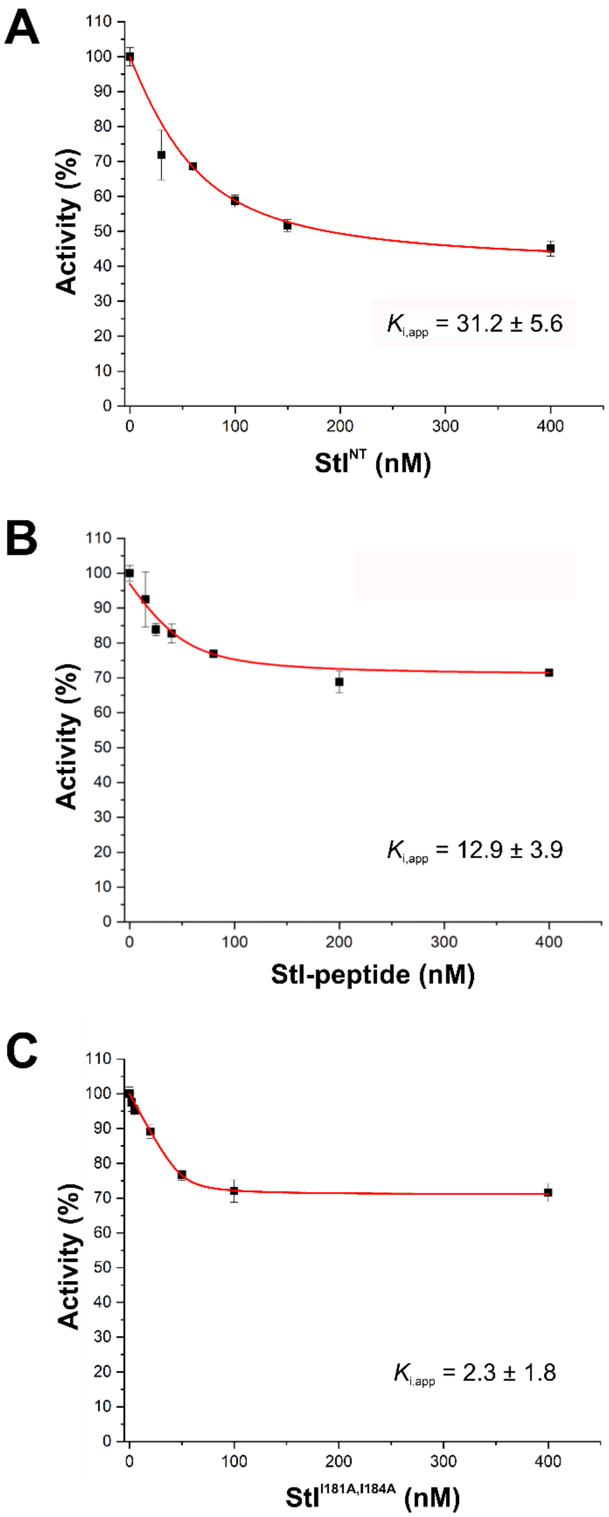


**Figure S7. Activity of human dUTPase in the presence of Stl$^{NT}$ (Panel A), Stl-peptide 95-DKMYSYVNKAYYNDGDIYYSSYD-117 (Panel B) and Stl-I181A,I184A (Panel C) protein variants.** Apparent inhibitory constant values ($K_{i,app}$ (nM)) are indicated on each panel. Individual panels were created with OriginPro 2018 (OriginLab Corporation) and the figure was assembled using CorelDRAW 2020 (Corel Corporation; https://www.coreldraw.com).

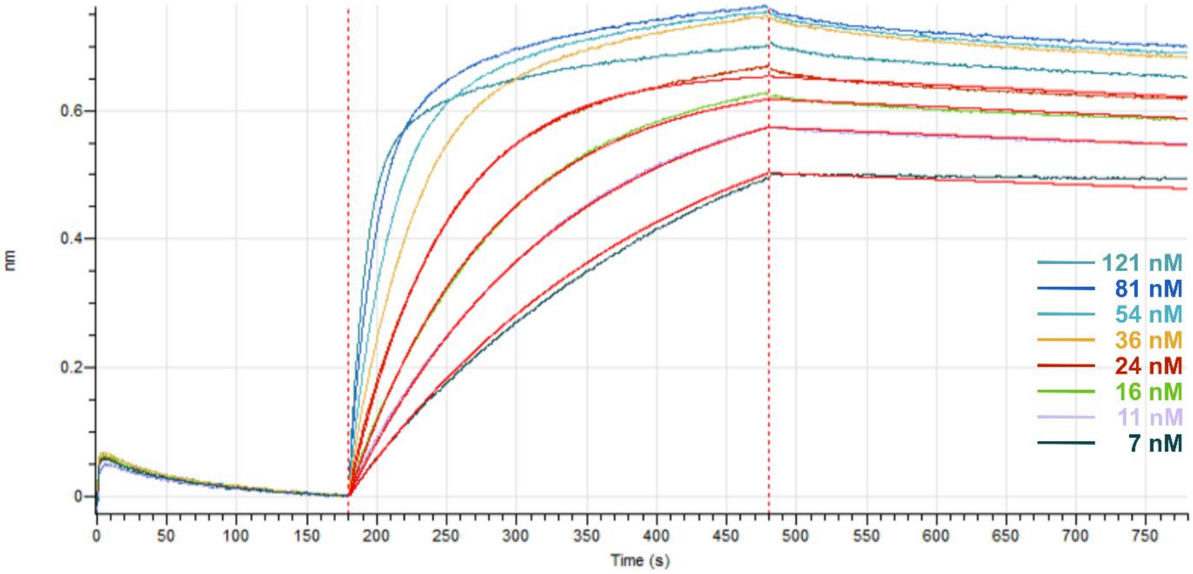


**Figure S8. BLI curves of Avi-tagged Stl$^{NT}$ and hDUT.** A 1:1 binding model has been fitted to the binding data indicated by red curves.

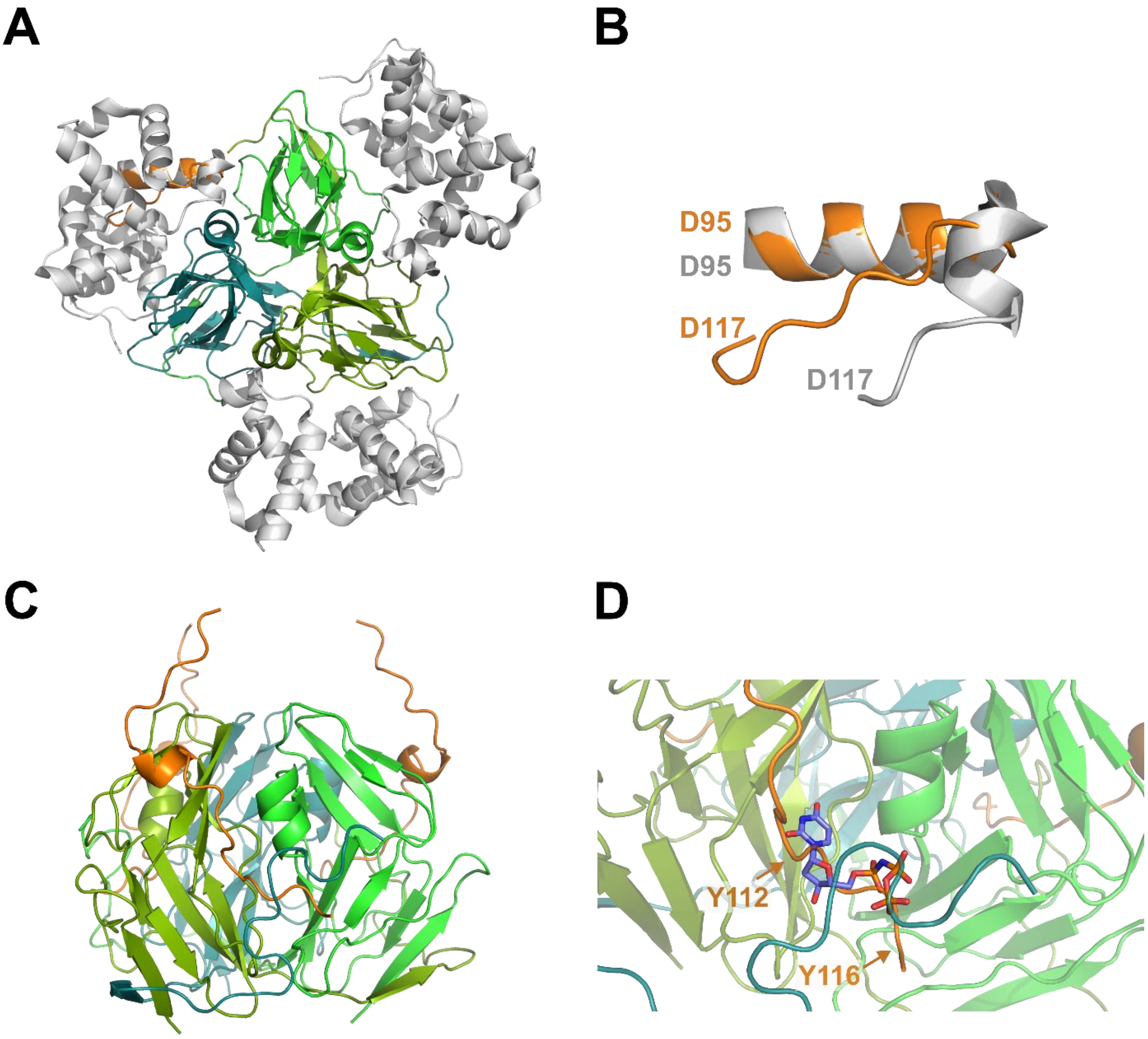


**Figure S9. Prediction of Stl-peptide structure and its complex formation with human dUTPase.** A) Structure of Stl-peptide (95-DKMYSYVNKAYYNDGDIYYSSYD-117) predicted by PEP-FOLD 3 superimposed to hDUT: Stl$^{NT}$ complex structure (PDB ID: 8C8I). hDUT trimer is represented as green and turquoise colored cartoons (different shades for the protomers), Stl$^{NT}$ is represented as grey cartoon, Stl-peptide is represented as orange cartoon. B) Comparison of the predicted and the experimental Stl-peptide structures. The representation of the PEP-FOLD 3 predicted Stl-peptide is the same as on Panel A, the corresponding segment of Stl$^{NT}$ from the 3D crystal structure (PDB ID: 8C8I) is shown as grey cartoon. C) AlphaFold3 based prediction of complex formation between Stl-peptide and hDUT. The representation of hDUT is the same as on Panel A, Stl-peptide in complex is shown as orange cartoon. D) Interference of Stl-peptide with substrate-binding. The dUPNPP substrate analogue is shown as sticks based on the structural alignment with the ligand-bound hDUT structure (PDB ID: 3EHW). The active site-interacting segment of the peptide is indicated by arrows and the beginning and the end of this segment. The representation of hDUT and the peptide is the same as on Panel C. Individual panels were created using PyMOL 2.5.4 (Schrodinger, LLC; https://www.pymol.org/) and the figure was assembled using CorelDRAW 2020 (Corel Corporation; https://www.coreldraw.com).

## Supplemental table

**Table S1. Data collection and refinement statistics**

| Structure | **hDUT:Stl$^{NT}$** |
| --- | --- |
| PDB ID | 8C8I |
| **Data collection** | |
| Space group | $P2_12_12_1$ |
| Cell dimensions | |
| *a*, *b*, *c* (Å) | 78.45, 82.66, 139.6 |
| α, β, γ (°) | 90.00, 90.00, 90.00 |
| Resolution (Å) | 52.15 - 3.20 (3.31 - 3.20) * |
| $R_{merge}$ | 0.2253 (1.115) |
| $I$ / σ$I$ | 9.60 (2.55) |
| Completeness (%) | 99.92 (100.00) |
| Redundancy | 13.0 (13.8) |
| | |
| **Refinement** | |
| Resolution (Å) | 52.15 - 3.2 (3.314 - 3.2) |
| No. unique reflections | 15556 (1530) |
| $R_{work}$ / $R_{free}$ | 0.1936 (0.2194) / 0.2448 (0.2806) |
| No. atoms | 6111 |
| Protein | 6110 |
| Ligand/ion | 1 |
| Water | 0 |
| *B*-factors | 72.16 |
| Protein | 72.15 |
| Ligand/ion | 77.04 |
| Water | - |
| R.m.s deviations | |
| Bond lengths (Å) | 0.005 |
| Bond angles (°) | 0.73 |

* Values for the highest resolution shell are shown in parentheses.
